# Integration of Digital Technologies into Goat Management in North-Eastern Kazakhstan

**DOI:** 10.3390/ani16193060

**Published:** 2026-09-29

**Authors:** Shaikenova Kymbat, Shauyenov Saukymbek, Assanbayev Tolegen, Sadenova Miram, Ibrayev Dulat, Omarova Karlygash, Mukhametzharova Ilmira, Doldasheva Gulzhainar, Sharapatov Tlekbol, Ismagulov Dalen, Aisar Mali

**Affiliations:** 1 Department of Animal Science and Veterinary Medicine, Saken Seifullin Kazakh Agrotechnical University, Astana 010000, Kazakhstan; k.shaykenova@kazatu.edu.kz (S.K.); s.shauenov@kazatu.edu.kz (S.S.); m.sadenova@kazatu.edu.kz (S.M.); d.ibrayev@kazatu.edu.kz (I.D.); k.omarova@kazatu.edu.kz (O.K.); i.mukhametzharova@kazatu.edu.kz (M.I.); g.doldasheva@kazatu.edu.kz (D.G.); highskillow@bk.ru (I.D.); 2 Department of Animal Science and Technology, Faculty of Agricultural Sciences, Toraighyrov University, Pavlodar 140000, Kazakhstan; asanbaev.tolegen.shonaevich@teachers.tou.edu.kz (A.T.); sharapatov.ts@teachers.tou.edu.kz (S.T.)

**Keywords:** precision livestock farming, digital technologies, Altai Mountain down goat, RFID identification, near-infrared spectroscopy, diet balancing, live weight dynamics

## Abstract

Precision livestock farming is increasingly applying digital tools to improve industry productivity and resource efficiency. This study evaluated the integration of a suite of digital technologies—RFID identification with electronic ear tags, the Sheep Auto Drafter automated weighing and sorting system, near-infrared spectroscopy (NIRS, FOSS DS 2500), and the “Korall” diet-balancing software—into the flock management system of 6000 Altai Mountain down goats under the agro-ecological conditions of north-eastern Kazakhstan. The chemical composition of six feed types was determined by NIRS: roughages contained up to 35.6% crude fiber and 35.6 mg/kg carotene; concentrate feeds provided up to 14.6% crude protein and 50.5% starch. Digital diets covered the metabolizable energy requirements of does (BW 34 kg) at 12.84 MJ/day and of bucks (BW 48 kg) at 16.86 MJ/day, in accordance with NRC (2007) norms. Over a two-month period 30 2.5-year-old does and 30 2.5-year-old bucks were monitored, and the live weight of the does increased by 4.2% and that of bucks by 5.3%, with average daily gains of 23 g and 42 g respectively. Assessment of exterior conformation and body indices of these 60 animals revealed pronounced sexual dimorphism: bucks had a significantly higher chest index (70.6% vs. 51.2% in does), while does showed a higher leg length index (51.2% vs. 43.8%). Digital diet analysis identified estimated dietary inadequacies relative to nutritional requirements of vitamin D, copper, and cobalt, justifying targeted premix correction. The results confirm the feasibility and potential benefits of integrating these technologies into goat management systems in Central Asia.

## Simple Summary

Sustainable goat production in pastoral regions often struggles with traditional, group-averaged feeding and labor-intensive manual record-keeping. This study evaluated an integrated digital flock management platform, combining rapid infrared feed testing, electronic ear tags, automated weighing platforms, and diet-formulating software. Over a two-month period, individual animal growth and body measurements were monitored from 30 2.5-year-old does and 30 2.5-year-old bucks. Rapid feed testing provided precise nutritional profiling of local forages, allowing the software to identify subclinical mineral deficiencies, particularly in vitamin D and copper. Digital diet balancing supported consistent live weight gains, with male goats growing faster than females due to natural physiological differences. Physical conformation analysis confirmed that body measurements develop largely independently, allowing for selective breeding without structural trade-offs. Overall, combining electronic identification, automated weighing, and digital diet calculation reduces manual labor, eliminates handling stress, improves weight tracking accuracy, and optimizes feed efficiency. This integrated digital approach establishes a practical, data-driven framework to enhance productivity, farm management, and economic sustainability for extensive goat farming across Central Asia.

## 1. Introduction

Goat farming is a traditional and strategically important livestock sector in the Republic of Kazakhstan. The country possesses extensive areas of inaccessible mountain (7.2 million ha) and stony (18.2 million ha) pastures that can be effectively utilized only through goat husbandry, forming a sustainable raw-material base for sectoral development [1,2]. The high adaptability of goats to extreme climatic conditions, together with their broad range of valuable products—down, milk, meat, and hides—drives growing demand both on domestic and international markets [3,4,5].

Goat products account for approximately 30% of total livestock output in European countries and more than 50% in Arab countries [6,7]. Young goat meat surpasses beef, pork, and mutton in nutritional value and digestibility, and goat fat is used in folk medicine for the treatment of respiratory ailments [6]. These characteristics underscore the strategic importance of goat farming for food security and income diversification of Kazakhstan’s rural population.

At the same time, the sector faces systemic constraints: unbalanced feeding rations, the absence of individual animal monitoring, and outdated selection methods. The concept of precision livestock farming (PLF)—including electronic identification, automated data collection, and decision-support software—opens transformational opportunities for overcoming these limitations [7,8,9]. The experience of France, the Netherlands, and New Zealand demonstrates that digital flock management systems can reduce production costs by 20% and increase profitability by 80–85% [9,10,11].

For Kazakhstan, the integration of PLF tools into goat husbandry is both technologically feasible and economically desirable. However, the peer-reviewed literature contains virtually no data on the application of integrated digital systems—NIRS feed analysis, RFID identification, automated weighing, and software diet balancing—under Central Asian conditions. The present study aims to fill this gap.

The research objectives were as follows: (1) to characterize the nutritional composition of local feeds by NIRS; (2) to develop sex- and age-specific rations using the “Korall” software; (3) to assess live weight dynamics using the Sheep Auto Drafter automated weighing system; (4) to evaluate exterior conformation parameters and body indices; and (5) to determine the overall effectiveness of integrated digital management.

## 2. Materials and Methods

### 2.1. Study Site and Animals

The study was conducted at a base farm located in the north-eastern low-mountain zone of Kazakhstan (50° N, 78° E; altitude 320 m above sea level). The total farm population comprised approximately 6000 Altai Mountain down goats kept under a semi-extensive management system that combined seasonal grazing with indoor housing. During the grazing season, the animals grazed for about 13 h daily and had regular access to water. In winter, they were kept primarily indoors; grazing time was reduced to 7 h per day, and their diet was supplemented with roughage and concentrates tailored to their physiological condition. This herd served as the source population for establishing the experimental sample used in the study. For the direct experimental study, 30 females and 30 males aged 2.5 years were selected from the total flock using the paired-analogue method. Animals were assigned to the experimental groups based on the following selection criteria: age (2.5 years), live weight, body condition score, origin (belonging to the same breed line), and similar management and feeding conditions at the start of the experiment. The formation of paired analogues made it possible to minimize the influence of individual animal variation on the study results and to ensure comparability between the experimental groups. All animal zootechnical procedures were conducted in accordance with institutional ethical standards; no invasive interventions beyond standard zootechnical procedures were applied.

### 2.2. Feed Analysis by Near-Infrared Spectroscopy (NIRS)

The chemical composition of six types of forage and feedstuffs (meadow hay, steppe grass, pasture grass, oats, oat–millet mixtures, and oat–sunflower mixtures) was determined at the accredited Kazakhstan–China Joint Laboratory of Biosafety of the Institute of Livestock and Veterinary Medicine. The evaluation was performed via near-infrared spectroscopy (NIRS) using a FOSS NIRS DS 2500 scanning monochromator analyzer (FOSS Analytics, Nils Foss Allé 1, DK-3400 Hillerød, Denmark). The instrument operated within the spectral range of 400–2500 nm, utilizing a silicon detector for the 400–1100 nm region and a lead sulfide (PbS) detector for the 1100–2500 nm region, with measurements recorded in reflectance or transflectance mode. The analysis time was less than one minute per sample.

The analyzer allowed for non-destructive rapid estimation of key nutritional parameters, including dry matter, crude protein, crude fiber, crude fat, ash, starch, neutral detergent fiber (NDF), and acid detergent fiber (ADF) contents, alongside the calculated metabolizable energy (ME) values. The concentration of macronutrients, specifically calcium (Ca) and phosphorus (P), was evaluated indirectly based on the mathematical correlations between mineral elements and the organic matrix of the studied plant tissues.

Instrument operation and spectral data processing were managed via the ISIscan Nova software (infofoss@foss.es), utilizing LOCAL regression algorithms and global artificial neural network (FOSS-ANN) calibration models. Feed samples were collected in layers from different feed batches, with five spot samples taken from each batch (*n* = 5 per feed type, *n* = 30 in total); samples were then transported to the laboratory in sealed packaging and analzed within 24 h of collection. Each sample was analyzed in three analytical replicates. The analytical accuracy of the local NIRS calibration models was confirmed by high coefficients of determination (R^2^cv = 0.92–0.95) and low standard errors of cross-validation (SECV = 0.38% for crude protein, 0.51% for crude fiber, and 0.32% for ash).

The analysis of diet balance and the identification of nutrient deficiencies in breeding bucks and does at the base farm were conducted prior to the experimental period using the digital “Korall” software (Korall-Agro, Moscow, Russia, https://www.korall-agro.ru/?utm_source=chatgpt.com (accessed on 17 September 2026)). The results of the chemical analysis of the feedstuffs under study were used as input data for the calculations. Thus, the laboratory-determined chemical composition parameters of the feedstuffs represented the experimentally measured input data, whereas the nutritional value of the diets and their provision with individual nutrients were determined computationally using the software.

The “Korall” program is a specialized digital tool designed for the calculation and analysis of diets for farm animals. Diet parameters were calculated taking into account the animal species and physiological category, live body weight, age, physiological status, and level of productivity. Based on the input data, the program calculated the dietary supply of energy, protein, amino acids, vitamins, and macro- and microelements and enabled the assessment of the adequacy of the calculated values relative to the animals’ nutrient requirements.

The nutrient requirements and dietary supply levels were compared with the recommendations of the All-Russian Research Institute of Animal Husbandry (VIZ, 2018) [12] and the National Research Council (NRC, 2007) [13]. The computational assessment of the initial diets using the “Korall” program revealed insufficient supply of certain nutrients, including vitamin D, copper, cobalt, iodine, and zinc. Based on the calculation results, balanced diet formulations were developed, and appropriate feed premixes were selected for use during the subsequent 60-day experimental period.

In the present study, the parameters obtained directly from the chemical analysis of the feedstuffs were considered experimentally measured data. In contrast, the indicators of the overall nutritional value of the diets, their nutrient supply, and the magnitude of nutrient deficiencies were calculated values obtained using the “Korall” program based on the laboratory data and the reference requirements provided by VIZ (2018) [12] and NRC (2007) [13]. This distinction allowed the directly measured parameters to be clearly differentiated from the calculated indicators used to assess diet balance and subsequently adjust the animals’ feeding regimes.

### 2.3. Digital Flock Management: RFID and Sheep Auto Drafter

Individual animal identification was performed using RFID electronic ear tags (134.2 kHz, ISO 11784/11785 standard) [14,15] read through radio-frequency panels. Live weight recording and animal sorting were carried out with the Sheep Auto Drafter automated system, equipped with load cells (accuracy ±0.1 kg), three-way sorting gates, and an integrated RFID reader. Data were transmitted to a central database integrated with the 1C: Accounting for Agricultural Enterprises in Kazakhstan software, ensuring real-time record-keeping.

The 60 experimental goats were weighed when they were 2 years 5 months and again at 2 years and 7 months of age (Δt = 60 days). Absolute gain (kg), relative gain (%), and average daily gain (ADG, g/day) were calculated from these data.

### 2.4. Methods of Taking Body Measurements and Body Composition Indices

Exterior assessment of 30 does and 30 bucks aged 2.5 years was conducted using a measuring stick and measuring tape. Seven standard measurements were recorded: height at the withers, height at the rump, oblique body length, chest width behind the scapulae, chest depth, chest girth, and cannon bone girth. Six body conformation indices were calculated from these measurements, leg length, elongation, compactness, massiveness, chest, and bone index, using standard zootechnical formulas. Animal body indices are metrics expressing the ratio (as a percentage) between anatomically related body measurements. They are used to characterize body conformation when assessing an animal’s exterior and constitution, allowing for a more precise description of the physical traits of the animals being compared. Key body indices include the following: the leg length index—the ratio of height at the withers minus chest depth to height at the withers (characterizing body proportions and limb development relative to the trunk); the elongation index—the ratio of the oblique body length to height at the withers (characterizing longitudinal trunk development); the compactness index—the ratio of chest girth to oblique body length (an indicator of body mass development); the massiveness index—the ratio of chest girth to height at the withers; the chest index—the ratio of chest width (behind the shoulder blades) to chest depth; and the bone index—the ratio of metacarpal (cannon bone) circumference to height at the withers (an indicator of skeletal development).

### 2.5. Statistical Analyses

To evaluate live weight dynamics and growth over time, a Two-Way Repeated Measures Analysis of Variance (Two-Way RM ANOVA) was applied, with Sex (does/bucks) as the between-subjects factor and Time (*t*_1_/*t*_2_) as the within-subjects factor, along with their interaction (Sex × Time). For morphometric traits and body conformation indices between sexes, independent-samples Student’s *t*-tests were conducted.

Prior to analysis, the data were tested for compliance with the assumptions required for parametric methods: normality of distribution was assessed using the Shapiro–Wilk test, and homogeneity of variances was assessed using Levene’s test. Where these assumptions were violated, the non-parametric Mann–Whitney U test was applied. For multiple comparisons, post hoc tests with Bonferroni’s correction were used.

Results are presented as M ± sem (mean ± standard error of the mean), with calculation of the coefficient of variation (CV, %) and 95% confidence intervals (95% CI) for mean values and for the difference between group means. To assess the magnitude of differences, effect size was calculated using Cohen’s d (small effect—d 0.2–0.4; medium effect—d 0.5–0.7; large effect—d ≥ 0.8).

Data processing was performed in R Studio (v. 4.3.1) using the stats, car, and effsize packages as well as in SPSS 26.0 for Windows [16]. Differences were considered statistically significant at *p* < 0.05; exact *p*-values are reported in the text and tables, along with the notations: * *p* < 0.05; ** *p* < 0.01.

## 3. Results

### 3.1. Feed Chemical Composition

The results of the NIRS analysis are presented in Table 1. Roughages (meadow hay, steppe grass, pasture herbage) were characterized by a CF content of 30.7–32.5% DM and high carotene concentrations (15.2–35.6 mg/kg natural weight), defining their key role as sources of fiber and provitamin A. These indicators are consistent with the findings of Laurent et al. [17], who demonstrated that the predominant feed type (pasture or hay) is the main determinant of carotenoid concentration in goat milk and tissues. NDF in roughages was 50–60%, ensuring adequate rumen buffering capacity.

Concentrate feeds (oat-based mixtures) showed higher numerical CP (11.5–13.1% DM) and starch (45.9–50.5% DM) contents, with an ME of 12.5–13.0 MJ/kg DM—approximately 40% higher than roughages. Pasture herbage showed the highest CP (14.6% DM) and carotene (35.6 mg/kg) contents, indicating the high nutritional value of diverse swards. These data are consistent with numerous studies demonstrating the positive effect of the concentrate fraction of the diet on the energy status and productivity of small ruminants [18,19,20].

### 3.2. Digital Diet Balancing and Identified Deficiencies

Based on NIRS data, diets for each sex–age group were formulated using three types of feed with the ‘Korall’ software (Table 2). The doe ration (total 2.31 kg/day as-fed) consisted mainly of meadow hay (20.49%), pasture herbage mass (59.35%), and oat (19.77%), providing 12.84 MJ ME/day—meeting NRC (2007) norms for lactating does of 34 kg BW. The buck ration (3.11 kg/day) covered 16.86 MJ ME/day in accordance with VIZh (2018) norms for bucks of 48 kg BW during maintenance feeding [12,13].

Despite adequate energy provision, the deficiency-flagging function of the “Korall” program identified potential micronutrient deficiencies in both groups (Table 2): vitamin D—9.9 mg/day compared with the recommended level of 17–25 mg/day; copper—1.73 mg/kg dry matter compared with the recommended level of 8–10 mg/kg dry matter; and cobalt—at the lower end of the recommended range (≈0.17 mg/kg dry matter compared with 0.2–0.3 mg/kg dry matter). In does, additional deficiencies in iodine, zinc, and vitamins A and E were identified, which may potentially impair reproductive performance and immune status [17,21]. These data clearly demonstrate the important role of digital diet balancing: traditional methods cannot simultaneously identify a range of potential dietary inadequacies relative to the animals’ nutritional requirements, particularly in extensive systems with variable feed composition.

To correct the identified deficiencies, the introduction of mineral–vitamin premixes (vitamin D, Cu, Co, I, Zn) was recommended. During the breeding season, supplemental feeding of bucks with carrots (carotene source) and eggs (source of biologically active substances) is advisable to stimulate reproductive function [12].

### 3.3. Live Weight Dynamics

Live weight dynamics of does and bucks over the 60-day monitoring period were analyzed using Two-Way RM ANOVA (Table 3). A significant main effect of Sex was observed (**F**_(**1**,**58**)_ = **5935.69**, ***p*** < **0.001**, $\eta_{p}^{2}=0.990$), with bucks being consistently heavier than does at both time points. A significant main effect of Time was also identified (**F**_(**1**,**58**)_ = **611.84**, ***p*** < **0.001**, $\eta_{p}^{2}=0.913$), confirming overall weight gain. Importantly, the Sex × Time interaction was highly significant (**F**_(**1**,**58**)_ = **48.67**, ***p*** < **0.001**, $\eta_{p}^{2}=0.456$). Over the 60 days, bucks gained an average of 2.5 kg (ADG ≈ **42** g/day), whereas does gained 1.4 kg (ADG ≈ **23** g/day).

### 3.4. Body Measurements and Conformation Indices

Evaluation of body measurements and conformation indices in 2.5-year-old goats revealed pronounced sexual dimorphism (Table 4). Independent-samples ***t***-tests showed that bucks significantly exceeded does in withers height, rump height, oblique body length, chest width behind the scapulae, chest girth, and cannon bone girth (***p*** < **0.001**, Cohen’s ***d*** > **0.8**). Conversely, chest depth was significantly greater in does (***p*** < **0.001**, ***d***= **1.233**). Among conformation indices, chest index was substantially higher in bucks (***p*** < **0.001**), while does exhibited a higher leg length index (***p*** < **0.001**). Elongation and bone indices showed no significant sex-based differences (***p*** > **0.05**).

Pearson’s correlation analysis revealed sex-specific patterns of association among principal body measurements.

Pearson’s correlation analysis revealed distinct sex-specific patterns of linear association among principal body measurements (Table 5). Overall, both does (***n*** = **30**) and bucks (***n*** = **30**) exhibited weak-to-moderate correlations (|***r***| < **0.40**), indicating a high degree of independence among individual skeletal and exterior conformation traits.

In does, the strongest positive association was recorded between chest width (CW) and chest girth (CG) (***r*** = **0.329**), reflecting proportional thoracic development in females. Conversely, a moderate negative correlation was observed between chest girth (CG) and cannon bone girth (CBG) (***r*** = −**0.361**) as well as between oblique body length (OBL) and chest girth (CG) (***r*** = −**0.269**). Other linear dimensions, including height at the withers (HW) and chest depth (CD), showed negligible relationships with other measurements (***r*** = −**0.132** to **0.215**).

In bucks, a similar positive trend was observed between chest width (CW) and chest girth (CG) (***r*** = **0.268**). Height withers (HW) was positively associated with chest depth (CD) (***r*** = **0.210**) and cannon bone girth (CBG) (***r*** = **0.202**). Inverse associations were noted between oblique body length (OBL) and cannon bone girth (CBG) (***r*** = −**0.280**) as well as between chest width (CW) and cannon bone girth (CBG) (***r*** = −**0.244**).

The general absence of strong collinearity (|***r***| < **0.50**) across both sexes confirms that these body measurements represent largely independent morphometric features, justifying their individual inclusion in evaluating exterior characteristics and conformational growth in Altai Mountain down goats.

### 3.5. Effectiveness of Integrated Digital Management

The combination of NIRS feed analysis, the “Korall” program, RFID identification, and automated weighing forms a coherent PLF package that eliminates three key bottlenecks in goat farm management: uncertainty about feed nutritional value, the “invisibility” of individual productivity, and the data deficit for selection decisions. NIRS provides accurate, rapid feed analysis (within 24 h) at substantially lower cost compared with wet chemistry. The “Korall” program converts analytical data into specific feeding recommendations and identifies microelement deficiencies undetectable by traditional methods. The RFID system combined with the Sheep Auto Drafter enables individual productivity recording without labor-intensive manual animal restraint. Integration with 1C: Accounting enables real-time production cost calculation, closing the management loop from feed procurement to financial outcome.

This integrated approach aligns with best PLF practices in European countries [4,5,6,10] and demonstrates full reproducibility of these technologies under the agroclimatic and infrastructure conditions of Central Asia. Data from analogous systems highlight the potential for improving operational efficiency and record-keeping accuracy, providing a structural framework for precision flock monitoring [9,10].

## 4. Discussion

The integration of precision livestock farming (PLF) tools into small ruminant production represents a paradigm shift from traditional, group-averaged management to data-driven, individual-level monitoring [4,6,13]. Under the semi-extensive farming conditions of north-eastern Kazakhstan, where environmental fluctuations and variability in feed quality naturally constrain animal performance, the combined application of NIRS feed analysis, digital ration balancing (“Korall”), RFID identification, and automated weighing demonstrated the feasibility and potential benefits of integrating these technologies into goat management systems [2,4,9].

### 4.1. Nutritional Precision via NIRS and Digital Diet Balancing

Rapid chemical profiling of regional feeds via near-infrared spectroscopy (NIRS) provided essential baseline data that traditional wet-chemistry procedures cannot deliver on a routine, operational timescale [2,22,23]. The high crude fiber (CF 30.7–32.5%) and neutral detergent fiber (NDF 50–60%) contents observed in local roughages (meadow hay and steppe pasture) underscore their critical role in maintaining optimal rumen buffering capacity and acetate-to-propionate ratios in goats [17,22]. However, the software-driven evaluation of the initial rations revealed that despite meeting bulk dry matter and metabolizable energy (ME) requirements (12.84 MJ/day for does and 16.86 MJ/day for bucks), traditional pasture-based feeding concealed subclinical micronutrient imbalances [12,13,19].

Specifically, the automated deficiency-flagging function in “Korall” identified critical deficits in vitamin D (9.9 mg/day vs. required 17–25 mg/day) and copper (1.73 mg/kg DM vs. required 8–10 mg/kg DM) alongside borderline cobalt levels [12,13]. Copper and cobalt are vital cofactors for antioxidant enzymes (superoxide dismutase) and vitamin “B” _12 synthesis, respectively; their subclinical deficiency often manifests as unthrifty growth, impaired wool/down quality, and suppressed immune function without overt clinical signs [12,17,21]. These findings corroborate the previous assertions by Nikitina [17] and Cadero et al. [24] that decision-support software plays an important role in identifying hidden nutritional deficiencies in extensive feeding systems and enables timely, targeted dietary correction through premix supplementation before economic losses occur.

### 4.2. Live Weight Dynamics and Sexual Dimorphism

The live weight dynamics captured by the Sheep Auto Drafter automated system confirmed the efficacy of the nutritionally balanced rations over the 60-day monitoring period [13,14,25]. The highly significant main effects of Sex (F_(1,58) = 5935.69, *p* < 0.001, η_*p*^2^ = 0.990) and Time (F_(1,58) = 611.84, *p* < 0.001, η_*p*^2^ = 0.913), together with their interaction (F_(1,58) = 48.67, *p* < 0.001, η_*p*^2^ = 0.456), demonstrate that growth responses were strongly conditioned by sex-specific physiological capacity [21,26]. Bucks achieved nearly double the average daily gain of does (42 g/day vs. 23 g/day), driven by inherent physiological sex differences and higher concentrate inclusion in their ration [7,18].

The low standard error values associated with automated load-cell measurements (0.07 to 0.23 kg) highlight the superiority of PLF weighing platforms over manual methods [4,25]. Eliminating human handling stress during passage through the three-way drafter not only ensured high data fidelity but also prevented handling-induced weight loss, aligning with observations in mountain sheep flocks by Morgan-Davies et al. [25,26].

### 4.3. Morphometric Differentiation and Structural Traits

Morphometric evaluation revealed pronounced sexual dimorphism across nearly all linear body dimensions and conformation indices [27,28,29]. Bucks exhibited significantly greater skeletal frame dimensions (height at the withers 68.5 cm, chest width 27.2 cm, chest girth 86 cm) and an elevated chest index (70.6% vs. 51.2% in does; *p* < 0.001, d = 4.770), reflecting heavy thoracic development and muscular draft capacity [28]. Conversely, does displayed a significantly greater chest depth (40.8 cm vs. 38.5 cm; *p* < 0.001, d = 1.233) and higher leg length index (51.20% vs. 43.80%; *p* < 0.001, d = 1.618). A deeper chest cavity relative to overall frame size in females provides necessary abdominal capacity to accommodate gastrointestinal volume during gestation and peak lactation, a recognized adaptive feature in native Central Asian goat breeds [8,27].

### 4.4. Practical Implications and Systems Integration

The overarching benefit of the deployed PLF platform lies in the seamless integration of disparate technological modules [4,6,7]. Bi-directional data flow—linking individual RFID identification, NIRS feed chemistry, automated draft weights, and financial accounting within the 1C platform—closes the management loop [2,7]. As emphasized by Wang et al. [28], integrating heterogeneous PLF data sources mitigates labor constraints typical of extensive pastoral farming while establishing a robust dataset necessary for future predictive analytics and digital machine learning [30,31].

## 5. Conclusions

The present study demonstrates that the integration of digital technologies—NIRS feed analysis, the “Korall” program, RFID identification, and the Sheep Auto Drafter automated system—into the management of Altai Mountain down goat flocks is highly effective and promising for improving goat management systems under the conditions of north-eastern Kazakhstan. The principal conclusions are as follows:

– NIRS analysis confirmed that roughages provide sufficient fiber and carotene, while concentrates cover the energy and protein requirements of both sex–age groups.
– Rations formulated with the “Korall” program met ME norms for does (12.84 MJ/day) and bucks (16.86 MJ/day) but revealed estimated dietary inadequacies relative to nutritional requirements of vitamin D, copper, cobalt, iodine, and zinc, necessitating targeted premix correction.
– Automated weighing recorded live weight gains of 4.2% in does and 5.3% in bucks over 60 days, with an ADG of 23 g and 42 g respectively, confirming the high productive potential of the breed.
– Exterior analysis revealed statistically significant sexual dimorphism: bucks showed a substantially higher chest index (70.6% vs. 51.2%) and does a higher leg length index (51.2% vs. 43.8%); the bone index was identical in both sexes (~14.1%).
– Integrated digital management improved data collection accuracy, reduced labor inputs, and provided end-to-end linkage of feeding, growth, and exterior indicators to the farm’s economic outcomes through the 1C platform.

The results establish an evidence base for the digitalization of goat farming in Kazakhstan and the Central Asian region as a whole. Promising directions for further research include assessment of the long-term economic effect of PLF implementation and development of digital learning algorithms for predictive flock management based on the accumulated digital data arrays.

## Figures and Tables

**Table 1:** Chemical composition of farm feeds (NIRS analysis).

No.	Feed	Moisture %	DM %	CP %	Fat %	CF %	Ash %	Carotene, mg	Sugar %	Starch %	Ca %	P %	ME, MJ/kg	NDF %	ADF %
1	Meadow hay	15.2	84.8	10.3	2.5	32.5	9.50	15.2	4.6	—	1.15	—	8.00	60	38
2	Steppe grass	77.5	22.5	8.00	2.7	31.4	10.4	32.7	2.2	0.80	1.02	—	9.50	52	30
3	Pasture herbage	80.8	19.2	14.6	2.4	30.7	11.8	35.6	15	1.50	1.50	—	9.50	50	32
4	Oat	12.4	87.6	13.1	2.4	7.60	3.00	—	1.5	45.9	0.09	0.28	13.0	20	6.5
5	Oat + millet	9.40	90.6	12.9	3.1	6.90	2.80	—	1.6	50.5	0.07	0.23	13.0	20	7.0
6	Oat + sunflower	10.3	89.7	11.5	2.8	7.90	2.60	—	1.2	47.5	0.06	0.21	12.5	22	8.0

DM—dry matter; CP—crude protein; CF—crude fiber; Ca—calcium; P—phosphorus; ME—metabolizable energy; NDF—neutral detergent fiber; ADF—acid detergent fiber; “—”—not detected.

**Table 2:** Summary characteristics of digital rations and key nutritional indicators.

Parameter	Does (LW 34 kg)	Bucks (LW 48 kg)	Norm (VIZh 2018; NRC 2007)
Diet mass (kg/day)	2.310	3.110	—
ME (MJ/day)	12.84	16.86	12–13/16–18
CP (% DM)	~11.50	~12.00	10–12
NDF (% DM)	~48	~46	35–50
Vitamin D (μg/day)	Deficient	9.9 (deficient)	17–25
Copper (mg/kg DM)	Deficient	1.73 (deficient)	8–10
Ca:P ratio	Imbalanced	~2.2:1	1.5–2:1

LW—live weight; ME—metabolizable energy; CP—crude protein; DM—dry matter. Norms according to VIZh (2018) [12] and NRC (2007) [13].

**Table 3:** Two-Way Repeated Measures ANOVA for live weight dynamics for the 60 goats studied.

Source of Variation	SS	df1	df2	MS	F	*p*-Value	η^2^p
Sex (between-subjects)	5838.075	1	58	5838.075	5935.69	<0.001 ***	0.990
Time (within-subjects)	114.075	1	58	114.075	611.84	<0.001 ***	0.913
Sex × Time (interaction)	9.075	1	58	9.075	48.67	<0.001 ***	0.456

Descriptive summary: does **t**_**1**_: **33.5** ± **0.49** kg, **t**_**2**_: **34.9** ± **0.38** kg (gain: 1.4 kg); bucks **t**_**1**_: **46.9** ± **0.60** kg, **t**_**2**_: **49.4** ± **1.26** kg (gain: 2.5 kg). Note: *** ***p*** < **0.001**. SS = sum of squares; df = degrees of freedom; MS = mean square; $\eta_{p}^{2}$ = partial eta-squared.

**Table 4:** Sex Differences in body measurements and conformation indices for the 60 goats studied.

Variable	Unit	Does M ± Sem	Bucks M ± Sem	t	df	*p*-Value	Cohen’s d	Sig.
Body Measurements								
Height at the withers	cm	59.3 ± 2.9	68.5 ± 4.4	−9.423	58	<0.0001	2.433	***
Height at the rump	cm	62.5 ± 3.1	70.7 ± 3.4	−9.576	58	<0.0001	2.472	***
Oblique body length	cm	62.7 ± 3.3	66.3 ± 3.5	−4.072	58	0.0001	1.051	***
Chest width behind the scapulae	cm	20.9 ± 1.2	27.2 ± 0.9	−22.163	58	<0.0001	5.723	***
Chest depth	cm	40.8 ± 1.7	38.5 ± 1.9	4.775	58	<0.0001	1.233	***
Chest girth	cm	81.7 ± 2.4	86.0 ± 2.8	−6.313	58	<0.0001	1.630	***
Cannon bone girth	cm	8.4 ± 0.2	9.7 ± 0.5	−11.628	58	<0.0001	3.002	***
Conformation Indices								
Leg length index	%	51.2 ± 4.4	43.8 ± 4.6	6.266	58	<0.0001	1.618	***
Elongation index	%	105.7 ± 7.6	105.6 ± 8.6	0.048	58	0.9622	0.012	ns
Compactness index	%	130.3 ± 7.9	119.0 ± 7.0	5.842	58	<0.0001	1.508	***
Massiveness index	%	137.8 ± 7.8	125.6 ± 9.2	5.514	58	<0.0001	1.424	***
Chest index	%	51.2 ± 3.6	70.6 ± 4.4	−18.474	58	<0.0001	4.770	***
Bone index	%	14.1 ± 0.8	14.1 ± 1.2	0.038	58	0.9702	0.010	ns

Note: *** ***p*** < **0.001**; ns = not significant (***p*** > **0.05**). **n** = **30** per group. Cohen’s **d**: large effect ≥ **0.8**.

**Table 5:** Pearson’s correlation matrix for principal body measurements for the 60 goats studied.

Does (*n* = 30)							
Variable	Abbr.	WH	OBL	CG	CW	CD	CBG
Height at the withers	WH	—	−0.132	0.017	−0.023	0.134	0.135
Oblique body length	OBL	−0.132	—	−0.269	0.040	−0.119	0.215
Chest girth	CG	0.017	−0.269	—	0.329	0.068	−0.361
Chest width behind the scapulae	CW	−0.023	0.040	0.329	—	−0.177	0.204
Chest depth	CD	0.134	−0.119	0.068	−0.177	—	0.145
Cannon bone girth	CBG	0.135	0.215	−0.361	0.204	0.145	—
Bucks (*n* = 30)							
Variable	Abbr.	WH	OBL	CG	CW	CD	CBG
Height at the withers	WH	—	−0.076	−0.063	−0.194	0.210	0.202
Oblique body length	OBL	−0.076	—	−0.089	−0.079	−0.098	−0.280
Chest girth	CG	−0.063	−0.089	—	0.268	0.061	−0.198
Chest width behind the scapulae	CW	−0.194	−0.079	0.268	—	0.160	−0.244
Chest depth	CD	0.210	−0.098	0.061	0.160	—	0.085
Cannon bone girth	CBG	0.202	−0.280	−0.198	−0.244	0.085	—

Note: Values below the diagonal represent correlation coefficients for does; values above the diagonal represent correlation coefficients for bucks. All correlation coefficients were not statistically significant, indicating a high degree of independence among morphometric traits. HW = height at the withers; OBL = oblique body length; CG = chest girth; CW = chest width; CD = chest depth; CBG = cannon bone girth. **rn** = **30rn** = **30** ***p*** > **0.01**.

## Data Availability

The original contributions presented in this study are included in the article. Further inquiries can be directed to the corresponding author.
